# Barriers to access to the Norwegian healthcare system among sub-Saharan African immigrant women exposed to female genital cutting

**DOI:** 10.1371/journal.pone.0229770

**Published:** 2020-03-18

**Authors:** Vivian N. Mbanya, Laura Terragni, Abdi A. Gele, Esperanza Diaz, Bernadette N. Kumar

**Affiliations:** 1 Department of Community Medicine and Global Health, Institute of Health and Society, Faculty of Medicine, University of Oslo, Oslo, Norway; 2 Department of Nursing and Health Promotion, Faculty of Health Sciences, Oslo Metropolitan University, Oslo, Norway; 3 Unit for Migration and Health, Norwegian Institute of Public Health Oslo, Oslo, Norway; 4 Department of Global Public Health and Primary Care, University of Bergen, Bergen, Norway; Erasmus Medical Center, NETHERLANDS

## Abstract

**Introduction:**

Millions of women and girls have been exposed to female genital cutting (FGC). The practice of FGC extends beyond countries in Africa and Asia in which it is traditionally practiced. Women living with FGC in Norway have been reported to be in need of healthcare, but there is evidence of suboptimal use of healthcare services among this group, and we lack the women’s perspective about this problem. This study aims to explore the experiences and perceptions hindering access and use of the Norwegian healthcare system among sub- Saharan African (SSA) immigrant women exposed to FGC.

**Method:**

This qualitative research was conducted using purposive and snowball sampling to recruit thirteen SSA immigrant women in Norway previously exposed to FGC. Interviews were conducted from October 2017 to July 2018. The Interpretative Phenomenological Analysis method was used.

**Results:**

The findings indicate that women experience barriers both in reaching out to the healthcare system and within the healthcare system. Barriers prior to contact with the healthcare system include lack of information, husband and family influence on healthcare, and avoiding disclosing health problems. Barriers within the healthcare system include care providers with insufficient knowledge and poor attitudes of care providers.

**Conclusion:**

This study reveals multiple barriers to healthcare access that co-exist and overlap. This indicates that SSA immigrant women are ‘left behind’ in being able to access and use the Norwegian healthcare system. Therefore, appropriate interventions to improve access to healthcare should be considered in order to reach Universal Health Coverage, thus having a positive impact on the health of these women. Equitable healthcare should be reflected in policy and practice.

## Introduction

Migration to Norway has grown in the past two decades, particularly with immigrants from sub-Saharan Africa (SSA) [1]. Presently, 112,786 immigrants and their descendants from SSA countries constitute part of the total Norwegian population [1]. The Norwegian healthcare system is a tax-based system embedded with the principles of solidarity and equity [2]. The Norwegian General Practitioners (GP) are the backbone of primary healthcare; they are responsible for all initial assessment, investigation, and treatment of patients. They also are responsible for referring patients to specialist care [2].

All asylum seekers and immigrants with a legal residence permit are entitled to the same health services as Norwegian-born patients. Immigrants exposed to female genital cutting (FGC) are also entitled to receive the required healthcare and free treatment in health matters related to FGC [3]. The practice of FGC “comprises all procedures involving partial or total removal of the external female genitalia for no medical reason” [4]. Following FGC, women reportedly suffer from short term and long-term health risks as a consequence of the procedure. Some of these health risks includes pain, hemorrhage, infection, urinary retention and injury to the urethra, wound healing problems, problems with menstruation, sexual problems, psychological consequences, increased difficulties in labor and delivery, shock, human immunodeficiency virus (HIV), and death of the neonate and the women [5–10].

Some studies in Norway and abroad have shown the association between FGC and adverse obstetric outcomes, including episiotomy, prolonged labor, obstetric tears/lacerations, and difficult labor/dystocia [11]. However, some studies also indicated no association [12]. Following FGC, women can reportedly suffer from post-traumatic stress disorder, depression, loss of trust, and permanent lifetime tissue damage [8]. Four (Type I, II, III, and IV) forms of FGC exist and classified based on how the practice was done on an individual. The practice of FGC not only affects the lives of girls and women in the countries in which it is traditionally practiced, but it also affects the lives of girls and women living as immigrants in high-income countries [13]. According to the Norwegian Directorate of Immigration (UDI), many immigrant girls and women from FGC practicing countries may have undergone FGC upon arrival in Norway [14]. Presently in 2019, Norway has 47276 immigrant women from sub-Saharan African FGC practicing countries [15]. In 2013, 44,467 such female immigrants were residing in Norway, and half of them were estimated to have been subjected to type III (also known as infibulation the most severe form) of FGC prior to migration [16, 17]. In Norway, these women are offered reconstructive surgery—called de-infibulation—to alleviate some of the complications resulting from infibulation [18].

Based on literature and the official Norwegian policies (https://www.udi.no), those exposed to FGC and living in Norway, are required to receive information about the legislation that prohibits the practice, the health consequences, and healthcare-related to FGC. Women have the right to contact their general practitioner, midwife/nurses at the local medical center or school nurse. They can also contact the women’s or children’s clinic at their local hospital [3]. A GP must refer women to these specialized services. However, the question is, do these women receive and know where to get this information?

Despite having a good welfare system and measures [19], providing equitable healthcare services to immigrants remains a challenge to the healthcare systems in Norway, probably because of its multiethnic/cultural population [15]. SSA immigrants in Norway, reportedly face challenges, including system barriers and personal experiences that impede their access to healthcare [20]. Many factors reportedly influence health and health inequalities within a population. Inequities in access to healthcare exist, and access to healthcare is considered a social determinant of health [21]. Barriers to accessing healthcare—including the lack of cultural competence of healthcare professionals—are some significant factors that cause inequities in healthcare [21]. Social determinants of health could refer to social and economic factors within the broader determinants of health [22], or the experiences of historical trauma, discrimination, and racism, which may affect certain groups of people within a population to influence health and be responsible for healthcare inequities [23–26]. Addressing social determinants of health can improve health and reduce disparities in health and healthcare [22].

In understanding how different social factors do interact to influence health and health inequities, intersectionality has increasingly been applied in health system research, especially to understand and respond to health disparities. “Intersectionality is a research approach that helps researchers to deepen their understanding of inequity through better reflecting on the complexity of the real world [27]. “It promotes an understanding of human beings as shaped by the interaction of different social categories as race, ethnicity, migration, gender, class, in varied ways to disadvantage different people depending on their characteristics and contexts [27].” These interactions occur within framework of connected systems and structures of law, policies, governments, religion, and institutions [27]. The intersectionality concept provides a more specific form of pinpointing inequalities, in developing intervention approaches, and in ensuring results are relevant within particular communities [28]. With the thoughtfulness of the concept of intersectionality, it would be appropriate to understand whether certain predisposing factors among SSA immigrant women exposed to FGC influence their ability to access and use healthcare services in Norway. Factors such as women circumcision status, being a black African, being a woman, originating from regions with different cultural attitudes, and believing in traditional African healing practices can influence healthcare-seeking behaviors. These factors can significantly put women at a distinct disadvantage within Norwegian society. Additionally, FGC practices are rooted in culturally sophisticated traditions that influence the practice, so, it may require an ethical and culturally sensitive health and social service provision.

While Norwegian healthcare providers’ experiences have been reported, including lack of knowledge about infibulation with women living with FGC [18], the experiences of SSA women exposed to FGC with the Norwegian healthcare system has not been adequately investigated. Additionally, in Norway, existing evidence on women’s and care providers' experiences around FGC mostly orients towards maternity care delivery, with less attention dedicated to healthcare delivery in other settings or for other problems, as emotional and psychosocial well-being and barriers to healthcare [29, 30]. This study aims to explore the experiences and barriers impeding circumcised SSA immigrant women’s access and use of the Norwegian healthcare system, both for maternity care and non-maternal healthcare needs.

## Materials and methods

### Participants and procedure

This research study used qualitative methods to collect data. Interpretative phenomenological analysis (IPA) was used to provide a detailed examination of participants' experiences [31]. The IPA approach is suitable for understanding immigrant women's subjective experiences and perceptions regarding factors that hinder their access and use of the Norwegian healthcare system. Participants in this study were immigrant women exposed to FGC from sub-Saharan Africa, living in Norway. Purposive and snowball sampling techniques were used in the recruitment process. Immigrants and their descendants from SSA countries, as described previously [32], were identified through several established cultural networks, including faith-based organizations and cultural groups. Those identified were informed of the research objectives. The sub-Saharan immigrants with legal residence, at least 18 years of age, and willing to participate were included in the study. The identified participants also referred us to other women. Each new referral was explored, and thus, a total of 13 participants were identified. This study was conducted from October 2017 to July 2018.

### Characteristics of women

Participants were mainly migrants from different SSA countries (Sudan, Sierra Leon, Somalia, Liberia, Mali, Nigeria, Eritrea, and Senegal), with a combination of Muslims and Christians. Most of the women were between the ages of 20–50 years, and half of the participants had up to secondary school education, with some having attended professional courses. Most of the participants were employed; in kindergartens, nursing homes, cleaning companies, and a few owned private businesses or small shops. Some were unemployed. All participants had lived in Norway between 6 months and 12 years. The main reasons for migration were to seek asylum and family reunification. Among the 13 women, four were unmarried, but two of them had children. The rest of the women were married and had children.

### Data collection

Through an information letter, all participants were informed of the study objectives and details, giving them the possibility to reflect upon their participation without undue stress and pressure. All participants gave both written and verbal consent to participate. Once the participants accepted, initial contact was made, and the researcher kept in touch until the agreed appointment date. In order to ensure cultural sensitivity, there was a need for the term “female genital mutilation” to be replaced with “female genital cutting” in the interview guide. The term genital mutilation was not acceptable by most women, so this study used the term female genital cutting.

The women who agreed to participate in the study were interviewed in Norwegian or English. This took place in their home or selected time and place of their choice and convenience. Data were collected through a semi-structured interview, using an interview guide developed by the lead author. For most of the participants, the interview started with open-ended questions. Participants were asked to reflect on their perceptions, and experiences of the factors that hinder their access and use of the healthcare services for FGC related-health needs. The interview guide covered topics that focused on their visits to the GP, other healthcare services visited, knowledge of FGC healthcare services, access to FGC health information, and the general perception of the healthcare system. The guide remained flexible, allowing the participants to highlight additional issues of concern to them. The guide allowed the exploration of unanticipated themes. Field notes were maintained documenting the interviewer’s perceptions and interpretations during each interview to ensure trustworthiness. The duration of the interviews lasted for 45 to 75 minutes. All the interviews were audio-recorded with consent. Participants were told they could withdraw from the study at any time without justification and were assured of anonymity in the publication of data. The women were not paid for their participation but were provided refreshments. None of the participants withdrew from the study.

### Ethical consideration

All participants gave their consent before the commencement of the interview. Participants were also informed that the data would be used for publications and conference presentations and were assured that data would be anonymous. The Norwegian Regional Committee for Medical and Health Research Ethics (2016/799/REK Vest) and the Norwegian Social Science Data Services (NSD) approved this research study.

### Data analysis

The analysis included all participants and utilized different stages of the IPA framework [33]. It began with carefully reading each transcript for familiarity with its content. Each participants’ perspective was examined carefully for a unique context, based on the principles of the IPA idiographic approach, which aims to explore in-depth experiences, in particular, barriers to access and use of the healthcare services for FGC health-related problems. Secondly, line-by-line coding was applied, focusing on each participant's concerns. Thirdly, accounts were cross-examined by searching for repetition. Based on the in-depth analysis of a single participant, emergent subjects were grouped based on interrelations between words and thoughts. Super-ordinate themes from all the transcripts were compiled, and connections between emergent themes were identified. The themes were grouped based on the conceptual similarities to highlight important aspects of the participant's account. Super-ordinate themes were then developed based on emergent themes across transcripts (Table 1).

**Table 1:** Main themes and sub-themes.

Main themes	sub-themes	
1.Barriers prior to accessing the healthcare system	Lack of information	• did not know where to find information about services provided for FGC • unaware of the consequences of FGC
	Husbands and family influence on healthcare	• husband and family members won’t allow women to take a personal decision on healthcare • power imbalance • cannot complain when there is pain or bleeding during sex • cannot complain to avoid separation or divorce • cannot complain to avoid rejection by family • fear of disrespect of husband and family • feel unhappy but cannot complain • feel angry but cannot complain • frustrated but cannot complain
	Avoiding disclosing health problems	• ashamed • fear of being judged by care providers • fear of flashback • shy
2. Barriers in the healthcare system	Care providers insufficient knowledge	• unfamiliar about FGC case • care provider acknowledge lack of training • unable to help women • care provider does not know types of circumcision
	The poor attitudes of care providers	• disrespect • interrogation • no confidentiality • GP murmuring with other care providers • suspect patient of committing a crime • call child protective services on women to screen women's children at school • call police on women for questioning • asking intruding question • glanced at women with suspicion • doubt women • women feel ridiculed and humiliated

## Results

All of the women in the current study experienced circumcision before migrating to Norway. Some of these women were in doubt about the type of FGC performed on them, while others knew they were stitched entirely (type III). The reasons for FGC and those who circumcised the women were different for each individual. Women in the girl’s family (grandmother, mother & aunt) and a friend in one case initiated the process of FGC. In most cases, older women or traditional birth attendants performed FGC. The findings revealed that all participants in this study had undergone FGC before migration.

All the women had some health problems related to FGC. These included recurrent infections, bleeding, general pain, painful menstruation, loss of libido, sexual dissatisfaction, abrasion during intercourse, urine retention, reduction in sexual desire, psychological distress, and trauma. However, the women were unsure if FGC was the primary cause of painful menstruation, loss of libido, sexual dissatisfaction, and trauma.

## Barriers prior to accessing the healthcare system

### Lack of information

Most of the participants in this study stated that they were not familiar with the Norwegian healthcare system besides the primary healthcare and the GP, and this hindered their ability to navigate and access the health system. Apart from the GP, the women were unaware of services that offer FGC care and were uncertain whether to make appointments with the GP for FGC health-related problems. The women did not know that they could receive a referral to a specialist for psychosexual and psychological health needs and counseling. The women voiced expectation of being informed by the health system or government about the services available for FGC healthcare and the kind of treatment offered at the healthcare services.

“No one is telling us where we can find help for female genital cutting. They only tell the children in school, but not those that are not in schools. In the hospitals, there is nothing written about female genital cutting care and where to find help. I read on the UDI page that we should report any case and that we can visit women's clinics for help, without giving the description and details of the women’s clinic. Before moving here, I was in England, and there we could walk straight into U.K’s National FGC center and talk about our health issues for FGC. It is not the same here.”

(Ngozi, interview transcript)

Participants expected to be told of other services in addition to the GP. Women with psychological problems related to FGC did not know where to seek help and were unaware that they could receive a referral to see a specialist for their psychological problems.

“I have this problem that is troubling me inside. I cannot tell someone because I don’t know if it is a sickness or not. Because I suffer from sexual dissatisfaction, where can I go to seek help and to explain to for advice? This is causing me to be depressed and traumatized. I have not been to the hospital for my problem because I do not know if the service for trauma and depression is here in Oslo, and even if it is here, where can I find it. It is very difficult to know where mental health services are in Oslo. I don’t know where to start finding the hospital […].”

(Fatou, interview transcript)

However, some of these women have heard about de-infibulation when asked. The participants said they need detail information about the women’s clinic that is stated on the directorate of immigration webpage and about the availability of other services for FGC.

To some participants, a lack of health information about healthcare services for FGC was frustrating.

“[…]Because I do not know where to get information about female genital cutting, it is very frustrating […].”

(Helen, interview transcript)

### Husband and family influence on healthcare

Besides the scarcity of information, women stressed the relevance of their family as the main barrier to reaching out to healthcare services. Some women in this study professed that their family members influenced their decision-making in seeking healthcare for FGC health needs, especially de-infibulation. Women in this study did not seek de-infibulation for varied reasons. First, women reported that their husbands wanted to open the “vaginal passage” naturally. The participants stated that their men believe that they will eventually open up their wives' “vaginal passage” in due time and that if their wives seek help, especially for de-infibulation, it will make them less of a man. This idea was common among the Sudanese and Somali women, who reported that their husbands prevented them from seeking healthcare as stated that their husbands long to widen the “vaginal passage” by themselves.

“I am really suffering because during sex. It is very painful, and I will have severe bleeding. I cannot go to see the doctor because he will be very angry that I am insulting him of not being able to open me up. He sometimes says that a man should be strong enough and be able to open. If he cannot open, then he is considered as not being a “man.” He says that I should persevere.”

(Akifa, interview transcript)

The second reason reported was in fear of separation, divorce, or rejection by members of their families. Some participants said it was hard to seek help because they were afraid of domestic violence, divorce, and economic deprivation. Some of the women were frustrated and unhappy due to pain and bleeding during sexual intercourse but could not complain to their husbands for fear of rejection.

“The bleeding happened more than once during sexual intercourse. Every time he is about to penetrate, he pushes hard forward, and because of that continuous pushing of the penis, maybe he did damage the very sensitive tissue. I experience a lot of pain and infection. I dislike sex because, after 2 to 3 days when he comes back for sex, I experience more pain and even more than the first time [. . .] Up to now it is still paining me, and I am very upset about it. I even suggested to him for us to seek medical attention. He refused, just to prove that he is a man […] He refuses to put lubrication cream, and he pushes and pushes as if I am not a human being. I cannot complain because he may become angry and call for separation.”

(Mariatu, interview transcript)

Apart from the fear of separation and rejection, some women did not want de-infibulation as an option as women were disgruntled with the outcome and they expressed that after reconstruction (de-infibulation), the appearance of the vulva may not be pleasing to their partners. Women who had undergone de-infibulation procedures reported losing potential suitors. Women stated that men absconded from being betrothed to them because of the appearance of the vulva following reconstruction (de-infibulation). For others, their husband prefers to have sex when the vaginal passage is narrow.

“My husband insisted that I must not go to the doctor without his consent because he likes to have sex when it is tight” […], I cannot refuse to comply.”

(Sara, interview transcript)

Some women could not complain because they were respecting the tradition. However, the tradition some are referring to is expected of them by their family and the community.

“[…] Despite the pains and bleeding, I cannot complain because according to our culture he has the right over my body. The bible also says so. And for him being the sole provider to the family and for fear of losing my marriage, I cannot refuse to comply.”

(Sara, interview transcript)

According to some women, communication between them and their spouses was poor in matters relating to their health. A few women professed that they could not enforce preferences in sexual situations, to show respect to their husbands. Some women stated that when their spouses did not allow them to seek healthcare, they felt that their spouses were not concerned about their pains.

“[…]. He [husband] does not care when it concerns my health and even if I speak he [husband] does not take me seriously, so I keep quiet and stay alone at my corner because I don’t want to disrespect him since he is my husband and father to […].”

(Nora, interview transcript)

“[…]. My sickness is my burden, not his [husband] own. He minds his business and […] [then comes a “sigh” sound].”

(Aamina, interview transcript)

### Avoiding disclosing health problems

Some women refused to seek help, voicing a need to avoid disclosing health issues to the care providers or talking to others about their feelings. Their refusal was due to fear─ fear of blame and judgment by the care providers, fear of disobeying and rejection from family, and fear of flashbacks caused by FGC. Most of them were also shy and ashamed to disclose their health issues, especially those with psychosexual problems.

“This is inhuman because I am sick of the continuous pain and bleeding. I feel ashamed to discuss this with the doctor. They may laugh at me because it sounds disgusting that my husband wants to open it up by himself.”

(Mariatu, interview transcript)

“I have a lot of pain during intercourse because I was completely stitched up. They [those who perform the circumcision] did not cut everything, but they sewed and left only a small hole that my smallest finger cannot get into my vagina. I feel uncomfortable, especially when in the public toilet because people standing out of the toilet door cannot hear the sound of my urine. I feel they [those standing outside the toilet] automatically know that I am circumcised. This has caused me to have a phobia of urine retention. How can I explain this to the doctor? I feel very shameful to discuss this with a medical professional.”

(Aamina, interview transcript)

Women suffering from sexual dissatisfaction, and recurrent urinary infections were ashamed and shy to disclose to the care providers. In addition to the general shame of disclosing sexual dissatisfaction to the GP, shyness, fear, and stigma attached to FGC deterred women from presenting complaints for their gynecological issues and for urinary tract infections. Women who were suffering from a repeated urinary infection, though unaware of the primary cause, expressed that they were feeling shy and stigmatized to talk about it to the care providers.

“[…] All the time infection, infection […] how can I tell the doctor. I am ashamed to tell anyone. This is causing me to have a stigma. I cannot tell anyone, and I cannot go to the hospital, all because of infection, infection [repeated] all the time. I am tired of this infection in this [pointing to the vaginal area].”

(Aamina, interview transcript)

Some women complained that recurrent infection occurs with sexual intercourse, and the abrasion caused during sexual intercourse tends to cause itching and swelling around the genitals. Some stated that seeking healthcare meant exposing their FGC. Despite the good intention of de-infibulation to allow intercourse and to facilitate childbirth, some women refused de-infibulation, in fear that their vulva will look unpleasant following reconstruction (de-infibulation).

“I am so worried that I may not find the right husband or boyfriend. Each time we are together, and they realized that I am circumcised, they will go and never return, not even a call. To me, it seems as if Somali men are interested in women who are not circumcised. This is very traumatizing because I cannot bring back my original private parts [sexual organ]. I blame my parents.”

(Habiba, interview transcript)

Those women who had been de-infibulated were worried that they might not find suitors of their choice, and they said to have accepted de-infibulation because of the advantages it can offer. Two of the women, previously refugees in Sweden, had undergone de-infibulation in Sweden. The women said their boyfriends left them because the women's sexual organs looked ugly and “abnormal.” The women regretted having undergone the repair (de-infibulation). According to the two women, their friends encouraged them to do the procedure. The two women professed that they did it because their friends told them that following the repair, they would have no health concerns with reproductive health and emotional distress.

“[…] when I came to Norway, my friend was rushing to be open [de-infibulation] and one of my friends encouraged me to do that because it will ease the pains and the emotional or psychological distress. I was so happy to be re-opened. Once I got married, it was two years past, and my husband started cheating on me […] he finally left me for one of my friends. He told my relatives that my vulva is ugly, and the flesh is hanging everywhere. Until date, I feel very disturb and angry with myself for doing it. When I walk around my community and among people from my country, I am ashamed. Maybe he [husband] told some people.”

(Titi, interview transcript)

Some women were afraid to seek care or disclose health problems, as they feared that family and members of the community would gossip about them undergoing de-infibulation.

“Once I tell my mum or someone that I am going in for repair [de-infibulation], they will talk about me. They will gossip around that I am no longer a virgin because the doctor will insert things [referring to medical instruments] into my private part. Because of the gossip, men may refuse to marry me. One is expected to marry while still a virgin.”

(Akifa, interview transcript)

Avoidance of healthcare was also due to fear of judgment or blame for something others did to them. Some participants said the care providers asked them some intrusive questions.

“I need a hospital where I can visit without being judged. Everyone [population] is judging me for having been circumcised. This is something [FGC] I was not aware of. I am now the victim […]. When I told a white doctor that I was in the clinic to seek help because of my health problems caused by circumcision […]. The way he looked at me, I felt stigmatized […].”

(Astou, interview transcript)

## Barriers in the healthcare system

### Lack of FGC knowledge among care providers

The participants perceived that the healthcare providers were unfamiliar about FGC cases, especially those who experienced challenges during delivery. Women also supposed that healthcare providers might be lacking training, as the women professed that healthcare providers were busy looking into books and computers before treatment. However, some participants professed that some health providers did acknowledge that they lacked training and were unfamiliar with FGC cases. Some of the women felt that clinic staffs (at the maternity setting) lack the skills and experience during childbirth of women exposed to FGC. Three women talked of traumatic experience during childbirth and the doctors and nurses not knowing what to do. This experience caused them embarrassment, fear, and more stress.

“When I was about having my number 5 child […] the child was not coming out. In the “birth room” [delivery room], there was a serious problem in the birth room” because the doctors and the nurses were running there and there, walking and talking to themselves as if some serious problem is happening to me or the baby. The doctor and nurses were reading in a book and asking me questions at the same time. I was very afraid and stressed because I thought I was about to die. It was embarrassing.”

(Aamina, interview transcript)

Participants expressed that healthcare professionals seem to be lacking confidence because they constantly read from a book before asking questions relating to their FGC condition.

“I do believe that the doctors here [Norway] know nothing about FGC because when you are talking to them, they will be focusing on a book before asking you questions. I was in the hospital for pains and bleeding from genital tissue damage; the doctor was busy looking up stuff from a book and working on his computer. Finally, I left his office unsatisfied.”

(Akifa, interview transcript)

Again, some women professed that healthcare providers acknowledged not being familiar with FGC cases. In this regard, healthcare professionals, as attested by some women, were regarded as not being a potential source of support.

“I asked him some questions, and he did not understand what I was saying. I asked him if he knew “pharaonic” circumcision. Because I wanted to explain to him […]. He said he was not aware and was not familiar with circumcision cases. I was disappointed and discouraged to revisit the hospital. He was unable to help me, so why should I waste my time to consult again.”

(Astou, interview transcript)

“During my menstrual cycle, the blood does not flow easily. It clots in the vagina and when I go to the toilet, I see a big lump of blood. I sometimes get an abscess. This is called in Arabic “khiraj”. When I went to see the doctor, he told me that he does not know what I am talking about and he has never treated a case of FGC during his professional practice […].”

(Mariatu, interview transcript)

### The poor attitudes of the care providers

For some women, care providers are more concerned about the criminalization of the practice than their healthcare needs. Women perceived care providers’ attitudes are limiting healthcare access; because most of the women said they did not seek care to avoid excessive questioning from healthcare providers. The women said that they were being interrogated and were considered “suspects” by the care providers. Women perceived this as disrespectful. The women complained that healthcare providers questioned them about their intentions of subjecting their children to FGC, and about their traveling plans to Africa.

“[…]. When I finally visited my doctor […]. I realized that she was interested to know if my children were circumcised and if I intended to travel with them to Africa. She was not interested in my health needs. When I realized that she was not paying attention to what brought me to the hospital, I immediately left the hospital.”

(Lissa, interview transcript)

Women also complained that the care providers glanced at them with suspicion, and they felt ridiculed. The women stated that some of the questions raised conflicts between them and the care providers, thus leading to tension, mistrust, and poor relationships with the care providers. The women felt that this had a profound effect on the way women viewed their interactions with the GPs. Most of the women felt that the questioning from care providers might be adding to their “worries” caused by FGC.

“The doctors in Norway do not support but instead add to the worries of women with circumcision. Before I used to visit the hospital for circumcision health problems, but I stopped […] I do it my own way, and I manage it with my own medicine [referring to traditional treatment].”

(Fatiya, interview transcript)

Two women expressed that during one of their visits to the GP, the GP interrogated them about their holiday back to their home countries. On the same day of the GP visit, the police and the social workers came to their houses for questioning and to check whether their children had been circumcised. The women perceived this as being disrespected by the GP.

“I realized that each time I leave the hospital, workers from the child protective services would come after my children. They [child protective service officers] go to their school to check them, and they will come to my house to question me. I realized that the doctors are trying to implicate me by calling the child protective services to take my children from me. When I do not visit the doctor, I have my peace. We [with husband] decided that it is better to stay away from the hospital because the doctors and nurses are acting as the police.”

(Fatou, interview transcript)

Confidentiality was an issue for some of the women, as it also led to conflict and stress, especially when the clinic staff asked intrusive questions that other patients could overhear. The women further complained that they were being “showcased” to medical students for study practice because they said students come around with papers and books during healthcare.

“I rather stay with my problems than going to the hospital to see the doctor. When I go to the doctor, I come back unhappy […] they speak loud so that other staff will know that I am circumcised, and people stare at me as if I have committed a big crime. They murmur and call other officers to come and see me. They come around with papers and pen to learn on my body […].”

(Nora, interview transcript)

The women raised other issues as their children were taken from school for a medical check-up to determine if they were circumcised. Our participants said they would not like to speak to the healthcare providers about their health problems because they feel confidentiality is no longer guarantee. The women stated that the doctors are causing them to have more fear, stress, and discomfort, and women expressed the doctors are more concerned with the criminal aspect of FGC while disregarding their well-being.

“The doctors are creating a big problem because people go to the doctors as a place of trust and confidentiality, but if they doubt us again and again [. . .] why should I go to the doctors if I do not trust him or her anymore. This is really huge damage to the women, and sadly, it is happening. The doctors are not supporting us in any way; rather, they are looking for someone to report to the police.”

(Habiba, interview transcript)

Women perceived that health providers are victimizing women exposed to FGC, perhaps because of the overwhelming attention of its illegality.

“[…] She [midwife] asked me many questions. She was making as if I have committed a crime […].”

(Lissa, interview transcript)

All of the women expressed feelings of judgment for having undergone FGC. The women expressed that this practice was performed based on the decisions of others and without their consent. The women felt ridiculed by the healthcare providers and the population as if they did this to themselves.

“Everyone [meaning population] is judging me for having been circumcised […]. […] I felt stigmatized. She [the doctor] glanced at me as being abnormal. I felt ridiculed. This was very annoying and made me unhappy and I had the feeling of emptiness.”

(Astou, interview transcript)

The women considered themselves to be the victims of circumcision and wished to avoid judgment. The women expressed that the doctors and the government feel that because they are circumcised, they might do it to their children. The women complained of undergoing many interrogations. The women believed Norwegian culture criticized them for an act they did not commit. The women said they prefer to stay in pains and isolation, rather than to face fear and humiliation.

“I am not a criminal and I know that circumcision is bad and I will not dare to circumcise my children. It is time for the Norwegian doctors and the government to change their perception about us especially those from Africa.”

(Fatiya, interview transcript)

“I will stay at home with my pains […] and it is better than going to the hospital and later come back in fear and disgrace […].”

(Helen, interview transcript)

## Discussion

Our study explored the views of 13 SSA immigrant women exposed to FGC on barriers to healthcare (maternal and non-maternal care), for FGC health needs. This paper specifically highlights the factors that impede women‘s access and use of the Norwegian healthcare services for FGC healthcare-related needs. SSA women exposed to FGC are facing challenges that impact their ability to seek care for FGC related maternal and non-maternal healthcare needs. Using the concept of intersectionality, we were able to understand the factors that influence SSA circumcised immigrant women’s ability to access and use healthcare services in Norway. The findings of the study revealed that women face barriers in and out of healthcare services. Barriers to access to healthcare were classified into two major themes: Barriers prior to accessing the healthcare system, and barriers in the healthcare system.

### Barriers prior to accessing the healthcare system

Lack of information on healthcare services and difficulties in the navigation of the healthcare system may not only be a challenge for women exposed to FGC alone but to other SSA immigrants in Norway [20]. The structure of the healthcare system in Norway could be an issue because most of the women mentioned that they were unfamiliar with the healthcare system in Norway. This might be different from what the participants have previously been exposed to while in Africa, thus making it difficult for them to navigate and use the Norwegian healthcare services. Another possibility is that the information about the healthcare system might be available, but a language barrier or health literacy could be a hindrance to some participants. Some women may not be able to read and comprehend available health information, including the kind of treatments offered at different healthcare services, thus causing a slow in the flow of healthcare information. In addition to lacking healthcare information, women require information on services that provide counseling, for psychological and psychosexual needs. Women in this study reported painful sexual intercourse (dyspareunia) and abrasion during intercourse, and this is higher with type III [34]. Although none of our participants reported having AIDS, theoretically, abrasion of the skin is the risk of transmission of HIV. Sexual intercourse with a circumcised woman is conducive to an exchange of blood, and FGC can correlate with a high incidence of AIDS [34]. Lack of sexual satisfaction and pains during intercourse was perceived to be the prime cause of psychological and psychosexual well-being. Therefore, educating women on the mental health consequences of FGC, and how to address such effects is vital to influence women to seek care for their psychological health needs. FGC can be a traumatic experience that may have both immediate and prolonged negative psychological consequences [11]. The psychosexual and psychological implications of FGC should be a priority to achieve health equity, as seems to be a shared sentiment among circumcised women, as has been reported in Iraq as well as in Kenya. [35, 36]. Importantly, good sexual health is fundamental to an individual’s health and happiness, for it could positively impact one’s reproductive health and well-being [37]. Having information about the availability of existing healthcare services and about the psychological consequences of FGC could influence a positive encounter with the healthcare system. In this respect, a high health literacy index among circumcised women could increase their ability to obtain, process and understand health information and healthcare services in Norway [38]. While linguistic barriers can hinder access to healthcare, reduce the quality of care, and result in dissatisfaction [39, 40], health literacy as a concept empowers health communication and stimulate understanding of the process of health communication in both clinical and community settings [38].

Husband and family influence over women was one of the main barriers for women to reach out to healthcare, especially for de-infibulation. The basis for women's husband refusing de-infibulation involved the husbands’ sexual choice of wanting a narrow “vaginal passage” and willingness to open the “vaginal passage” naturally and as perceived for “husbands sexual enjoyment.” “Male sexual enjoyment” reportedly attributed to the continuity of FGC practice, and women in Africa reportedly depend on their husband’s consent to seek healthcare, irrespective of their health needs [41]. The family refusal was possibly for cultural reasons. The husband's willingness to open the “vaginal passage” naturally might be that their husbands may either want to face their family with courage, face their friends and community with the pride of fulfilling and accomplishing their marital duties and responsibilities. The “natural way of opening” might be partly responsible for the recurrent infections, bleeding, and pains during sexual intercourse, as also perceived by the women.

Similarly, circumcised Somali women in the UK did not seek de-infibulation but had opted for their husbands to “open” the vaginal way naturally [42]. However, this was the reverse for women in the present study. This result could explain the reason for the unpopularity of de-infibulation among circumcised Somali women in the UK [43].

The participants who refused to undergo de-infibulation in the present study were immigrant women from Somalia and Sudan. It would be essential to look at de-infibulation among circumcised women from these ethnic groups, for family influence may be limiting women wanting to undergo de-infibulation. The result could also explain the reason behind the findings of a previous study on the experiences and management of birth care among women exposed to FGC in Norway, where health providers expressed concern about the birth care of circumcised women because they were mostly infibulation [18]. However, some of the participants seem to be influenced by their culture because it seems like the women themselves consider the natural version of the vulva as not aesthetically optimal. Some women were disgruntled with the outcome of de-infibulation.

Some participants refused to seek healthcare in fear of divorce, separation and family rejection. Women may be afraid of stereotypes of unmarried women, which results in stigmatization and marginalization [44, 45]. SSA African women have reportedly experienced this based on their marital status [46]. Again women in fear of family rejection could very likely be the associated risk and outcomes of family rejections [47]. One of the core fabrics of African customs is ‘respect’ and disrespect—especially of the elderly—and absence of respect is considered a misgiving of the young person [48, 49]. A husband maintains a strong influence as the head of the family [50], and this may be a justification for why some women could not enforce preferences in sexual situations, to show respect to their husbands.

Another factor that hindered healthcare was the fact that women were avoiding to disclose their health problems. Women were either shy or ashamed to reveal health problems, especially those related to sexual needs. As mentioned, they were in two minds between keeping it to themselves and consulting a care provider. This feeling was particularly communicated by women who experienced recurrent urinary infection and those experiencing psychosexual problems like loss of libido and sexual dissatisfaction. Women with FGC are reportedly likely than women without FGC to experience urinary tract infection and pain during intercourse [51]. Our study negates the hypothesis that FGC causes psychosexual problems to circumcised women [36]. Women in our study may refuse to seek care because they may not want information about their FGC status to be public [52]. Another reason might be that many female community members might have experienced similar health complications such that certain FGC related symptoms have become “normalized” and women might find it not relevant to consult a health-care provider [52]. Another reason for not disclosing health issues was in fear of judgment or blamed by the healthcare providers for their circumcision status and being blamed by their community for seeking de-infibulation. For this reason, the community may negatively impact women's healthcare, especially if the community members gossip after a woman seeks de-infibulation. As perceived, this is also critical for women because when gossip is circulating in the community, they risk the chance of losing potential suitors.

### Barriers in the healthcare system

In addition to barriers prior to accessing healthcare services, women also experienced challenges in the healthcare system that impede them from using healthcare services. Women in our study attested that the criminalization of FGC practice might override their health needs. The participants professed concern that healthcare professionals are more concerned with the illegality of FGC practices than their health needs. Not only did the women express this concern, but they were also equally worried about their feelings/emotional states.

Most of the women believed that the healthcare system is prejudiced, and according to most of the women, the healthcare providers were asking intrusive and interrogating questions at healthcare. Women perceived this as discrimination, lack of support, and disrespect. According to the women, these questions cause fear, trauma, doubt, mistrust, and becoming a “suspect” and vulnerable. They also said these questions aimed to ridicule them. Healthcare professionals need to be cautious because circumcised women may perceive the questions differently. As documented in other countries, African women exposed to FGC had experienced humiliation and women avoided questions from healthcare providers that triggered recollections [42, 53]. Nevertheless, according to Klein DC (1991), although the feelings associated with humiliation are strongly personal, the process itself exists in the link between the person and “the emotionally relevant human environment”[54].

The women perceived that the personal questions pointed to their race and countries of origin. However, there have been mixed opinions around FGC prevention in the healthcare setting and some circumcised women have argued that FGC prevention is needed in the healthcare setting. Still, it should be done without causing offense [55]. Creating a pleasant atmosphere during healthcare would encourage trust [56], and enable FGC patients to open up the discussion about FGC, and possibly influence revisit to the healthcare services.

Not only did the women had these experiences at the healthcare settings, but the findings also revealed that women had unannounced home visits (after a hospital consultation) by social workers and police. These were perceived to be uncomfortable, fearful, traumatizing, and adding to their worries caused by FGC. The women believed the system did not trust them, and as a result, the women did not trust the system. Such experiences negatively influenced the participants’ ability to access healthcare. However, as mentioned by Fangen K, in her study, many Somali in Norway feel intensely humiliated by the way they are met by public officials [57]. This result may partly explain the women’s feelings in our study when approached by public officials. Impromptu home visits of circumcised women in the UK have also been reported to frightened women and upset girls when interrogated in schools about their traveling plans [55]. Importantly, women in our study professed that they are aware of the laws abiding FGC, and they will not, in any circumstances, subject their children to the practice.

Most countries across the globe (with a few exceptions) recognize FGC as a violation of the human rights of women and girls. Several international rights treaties support the right of physical integrity and freedom from all forms of torture, degrading treatment, and discrimination [58, 59]. In Norway, not only does the government regards the FGC of girls as a crime against children, but it also recognizes FGC as a violation of human rights [60]. In 1995, the Norwegian parliament passed marked laws against FGC, with several measures in place to address and fight FGC. The women in our study are aware of the legal implications of FGC, so emphasizing it during healthcare would not only ruin a patient-care provider relationship, and cause mistrust, but also impact women’s’ subsequent visits to healthcare services. Some authors have documented that the laws and policies preventing FGC in high-income countries might have a negative influence on the abilities to access and use the healthcare system of those affected in the host countries [55].

Further, breach of their privacy and lack of confidentiality in healthcare as perceived by the women impedes the women’s ability to access maternal health services. This breach causes tension and even distrust between the healthcare providers and the women. A breach of confidentially, according to the women, caused stigma and created an atmosphere of fear and feeling of suspicion. This atmosphere could negatively affect women’s subsequent visits to health centers. According to McCartney, in 2015, disrespecting confidentiality is not the answer to FGC [61]. Breach of confidentiality utterly destroys patients’ trust in health services and stigmatizes patients further [61]. Perceptions of mistrust of care providers from Somali women patients and their families reportedly cause resistance to obstetric interventions [62].

The last but not the least of the factors that impede women’s ability to seek care were healthcare providers' awareness and knowledge about FGC. This factor is essential in providing adequate care because women exposed to FGC have professed a greater satisfaction and comfortability in discussing FGC with health workers with prior knowledge of FGC [63, 64]. Not only have healthcare providers acknowledged communication challenges with FGC patients and a lack of formal training or protocols guide for FGC [56], there have been studies that acknowledge poor knowledge regarding different aspects of FGC among healthcare professionals [65]. However, some studies emphasized that healthcare professionals may require the confidence to talk about the subject due to insufficient knowledge, may lack the experience in handling patients with FGC, or may lack understanding of patient culture [53]. For this reason, an understanding of the socio-cultural background surrounding FGC practice is crucial for healthcare providers to improve FGC management [66, 67].

### Strength and limitations

Our study has strengths and limitations. Our qualitative research, as far as we are aware, is the first to describe how SSA African women exposed to FGC experience and perceive healthcare in Norway. An advantage of our research study is that it was planned and designed by a team of immigrant professionals with research experiences in community, public health, and social science. Our team has carried out extensive research on immigrant's health and well-being in Norway. Each team member provided guidance based on his or her professionalism, from the planning phase to the design and data analyses, thus assuring the richness and quality of the data.

The interviewer’s background as a female African immigrant created a relaxed atmosphere. The women considered the researcher as one of them, so there was a strong relationship built on trust and mutual respect, and this might have encouraged open and honest responses. It may also be possible that some participants would have downplayed some negative experiences to avoid criticizing their husbands, the healthcare professionals, and the government in front of the researcher. However, as seen from the results, this was relatively small because the participants reported an in-depth range of their experiences from every viewpoint. To overcome the challenges of recruitment—especially as this group of women is hard to reach—friends known to be circumcised were recruited other women exposed to circumcision. Consequently, the variation in the group of women recruited might have been limited.

The insight gained from our study may be valuable when considering optimizing healthcare for sub-Saharan African women exposed to FGC. However, a limitation is not being able to capture any health professionals’ viewpoints. Interviewing through triangulation methods would have then been possible. Similarly, it would also be an advantage to interview husbands and men, but this view was beyond the scope of this study.

### Recommendations

The recommendations here originate from what women said and what has been shown in the literature to be significant in improving women’s access to healthcare and issues surrounding circumcised women’s health. Despite legislation discussing FGC as a violation of human rights, the health needs of those exposed to the practice are overshadowed by the legislation to safeguard FGC practice. Healthcare professionals need to find a way to bridge the void created after FGC, in that healthcare providers must provide excellent support to the women. Patients expect that all healthcare professionals should identify and report concerns about girls at risk of FGC. However, it is equally crucial that they inspire women and girls to seek healthcare for their FGC related-health problems. Support from healthcare professionals to women exposed to FGC is vital for a positive encounter with healthcare services. Because FGC has been high on the agenda in Norway, these women are fully aware of its ethical and criminal implications. Our study suggests that it would be necessary for healthcare professionals to ensure that a reasonable risk is identified, before contacting the child protective services. Healthcare professionals must be aware that many immigrant women exposed to FGC become fearful and worried when seeking medical care in their host countries [68]. Therefore, creating a good relationship and an environment of trust with the patients would lessen their fear and give room for positive outcomes [68]. It is essential to explore further the issue of criminalization and its impact on women's healthcare since our data may not adequately provide all aspects of the evidence.

It is important to create awareness among women exposed to FGC regarding seeking help for their health needs and where and how to get help through community-based educational programs [63, 68]. Education to women and care providers may be complementary and equally useful to encourage women who are shy or ashamed of presenting their health problems to come forward and seek help. Women exposed to FGC need social support networks for guidance and to provide stability to overcome some of the stigma associated with FGC. Community support may change the views and the perceptions of other community members about FGC. A well-functioning referral system and a good social support network play a key role in encouraging access to healthcare [69]. A good support network reportedly empowers women exposed to FGC to access antenatal and intrapartum services in England [42].

It would be important that healthcare professionals are respectful, non-judgmental, and open-minded when caring for women exposed to FGC. To foster a trusting relationship with women, healthcare providers must have a good understanding of the cultural background surrounding this practice [70]. According to Cindy Little, holistic care given within the context of culture is the most effective [68]. Social and healthcare professionals might need to reinforce their practice to reach an appropriate balance with regards to their legal obligations along with their fundamental responsibility to provide equitable and compassionate care to women.

To facilitate discussion about FGC concerns, care professionals' knowledge and attitudes to FGC—and a positive relationship with the patients—are essential [71]. Assessing care providers for knowledge about FGC is necessary for establishing whether additional training and guidance are required. The absence of specific guidelines may give rise to misunderstandings [72].

## Conclusion

Women exposed to FGC are subject to multiple forms of barriers to getting healthcare in Norway. Women also lack the necessary information, especially about the psychological and psychosexual consequences of FGC, and apart from the GP, they do not know where to seek help in Norway. Mostly, at different points in time, these barriers co-exist independently or interact with one another to impede access and use the Norwegian healthcare system. Importantly, women’s concerns and needs are not adequately addressed in the Norwegian healthcare system, leading to a circle of despair and surrendering to the inevitability of their hopeless situation. It is, therefore, important that these issues are adequately addressed by appropriate and relevant training of healthcare professionals and by information provided to the women to improve access to healthcare. Policymakers must address and prevent institutional discrimination issues and race-based inequalities in healthcare in Norway.

## Supporting information

S1 Text Interview guide for female genital cutting healthcare. (DOCX)
