# Supplementary material for: Barriers to access to the Norwegian healthcare system among sub-Saharan African immigrant women exposed to female genital cutting
Source: PLoS One. 2020 Mar 18;15(3):e0229770. doi: 10.1371/journal.pone.0229770 (PMC7080260; doi:10.1371/journal.pone.0229770)
Supplement: S1 Text — (DOCX) [file pone.0229770.s001.docx]

*The University of Oslo, Faculty of Medicine-Department of Community Medicine and Global Health*

**Interview guide for the healthcare of women exposed to female genital cutting (FGC)**

Date of interview

Participants name: N°:

Hour:

Duration:

Language of the interview:

Place:

Area:

**Socio-demographic characteristics**

Age: Home: Contact:

Religion: Education: Marital status: Number of children: Profession:

Country of origin:

**Perception of general health and decision to seek help for FGC**

- What is the perception of your general health? (researcher wants to ascertain the presence of ill-health)
- Do you have any health issues caused by female genital cutting? Or do you have some health issues which are caused by female genital cutting?
- What are your common health problems that are caused by female genital cutting?
- How do you feel when you have these health problems/ concerns? What do you do when you have these health problems/ concerns? Do you take care of them at home/ by yourself? Or do you seek help somewhere else?
- Why did you need help? When did you decide to seek help? How did you report them for healthcare?
- Do you know where to seek help for your particular health problems?
- Did you decide to seek help by yourself or did someone else decide it?
- Where have you been first with the health problems?

**FGC healthcare /healthcare services information**

- Are you aware of healthcare services that offer care for the kind of your health problems?
- How do you access health information pertaining to your health concerns?
- Did you require health information’s about the health condition?
- How/where did you get the health information’s
- Why didn’t you get information or advice about the health problem?

**Barriers to access to healthcare**

- Would you ever consult a doctor or other healthcare workers for female genital cutting health needs? Why?
- Have you been to the hospital or any other healthcare services for your health problems?
- Who attended to you on the day of the visit?
- Did you face any challenges, or had some difficulties in seeking healthcare for the health problems caused by female genital cutting?
- What are the issues (relating to female genital cutting) that make it difficult for you to access health care? What were your experiences with healthcare providers? How did you get about the difficulties? (Probe: Can you tell me more about it? (This was to allow the women to go in-depth into their challenges if any)
- Did you face a problem or had some difficulties navigating the healthcare system? Can you tell me more about that? (Probe: Researchers’ intention was to allow the women to reflect on the barriers to accessing the health care system and navigating the healthcare system).
- What are your experiences and challenges in visiting the general practitioner, emergency room, and other healthcare services for your health concerns caused by female genital mutilation? Please, can you tell me more about it?
- Can you tell me other factors that make it difficult in seeking healthcare for female genital cutting?
- Did you have some problems during pregnancy and childbirth caused by female genital cutting? What problems? Can you please tell me more about it? What were your experiences during childbirth?
- What are your beliefs in the care (e.g. advice, support) offered by the healthcare workers at the health facilities?
- What are other worries someone has caused you, not to seek help?

**Family involvement in healthcare**

- What is the level of approval/disapproval within your family to seek care for female genital cutting?
- Do you think your family would approve you seeking help for your health needs caused by female genital cutting?
- Who accompanied you to see the care professionals? Was your husband with you or any member of your family? What were your experiences in healthcare settings? Can you tell me more about it?

**Knowledge of FGC health consequences**

- Do you know or have an idea of the health consequences of female genital cutting?
- Do you think there is a relationship between female genital mutilation and psychological issues? Why?
- Do you think there is a relationship between female genital mutilation and sexual issues? Why?
- How common do you have sexual health problems caused by female genital cutting?
- How easy would you feel about consulting on sexual health issues?
- What are other worries FGC has caused you?

**Perception of the Norwegian health care system**

- Does the Norwegian health system meet your female genital cutting health needs? What are your experiences with the healthcare system in accessing the system for female genital cutting healthcare?
- What would you have changed in the Norwegian healthcare system?
- What do you like about the Norwegian health system

**Interviewer’s notes**

……………………………………………………………………………………….

……………………………………………………………………………………….

……………………………………………………………………………………….

……………………………………………………………………………………….

……………………………………………………………………………………….

……………………………………………………………………………………….

……………………………………………………………………………………….

……………………………………………………………………………………….

……………………………………………………………………………………….

……………………………………………………………………………………….

……………………………………………………………………………………….

……………………………………………………………………………………….

……………………………………………………………………………………….

……………………………………………………………………………………….

……………………………………………………………………………………….

……………………………………………………………………………………….

……………………………………………………………………………………….

……………………………………………………………………………………….

……………………………………………………………………………………….

……………………………………………………………………………………….

……………………………………………………………………………………….
